# Activation of TRPV1 by Capsaicin or Heat Drives Changes in 2-Acyl Glycerols and *N*-Acyl Ethanolamines in a Time, Dose, and Temperature Dependent Manner

**DOI:** 10.3389/fcell.2021.611952

**Published:** 2021-04-16

**Authors:** Meera Manchanda, Emma Leishman, Kishan Sangani, Ali Alamri, Heather B. Bradshaw

**Affiliations:** Bradshaw Laboratory of Lipid Neuroscience, Indiana University Bloomington, Bloomington, IN, United States

**Keywords:** TRPV1, lipidomics, thermoregulation, endocannabinoid, anandamide, 2-AG

## Abstract

Endocannabinoids (eCBs) and transient receptor potential (TRP) channels are associated with thermoregulation; however, there are many gaps in the understanding of how these signaling systems work together in responding to changes in temperature. TRPV1, a calcium-permeable ion channel, is activated by capsaicin, elevated temperature, the eCB Anandamide, and over 15 additional endogenous lipids. There is also evidence for signaling crosstalk between TRPV1 and the eCB receptor, CB_1_. We recently found that activation of TRPV1-HEK cells by capsaicin increases the production of the eCB, 2-arachidonoyl glycerol (2-AG), suggesting a molecular link between these receptors. Here, we tested the hypothesis that TRPV1 activation by capsaicin drives regulation of a wider-range of lipid signaling molecules and is time and dose-dependent. We also tested the hypothesis that changes in temperature that drive changes in calcium mobilization in TRPV1-HEK will likewise drive similar changes in lipid signaling molecule regulation. Lipid analysis was conducted by partial purification of methanolic extracts on C18 solid phase extraction columns followed by HPLC/MS/MS. Capsaicin increased the release of 2-acyl glycerols (2-AG, 2-linoleoyl glycerol, 2-oleoyl glycerol), in a concentration- and time-dependent manner, whereas levels of *N*-acyl ethanolamines (NAEs), including Anandamide, were significantly decreased. Analogous changes in 2-acyl glycerols and NAEs were measured upon ramping the temperature from 37 to 45°C. In contrast, opposite effects were measured when analyzing lipids after they were maintained at 27°C and then quickly ramped to 37°C, wherein 2-acyl glycerol levels decreased and NAEs increased. These results provide further evidence that the eCB system and TRPV1 have integrated signaling functions that are associated with the molecular response to temperature variation.

## Introduction

Temperature, touch, and pain are perceptions resulting from CNS processing of the transduction of stimuli by somatosensory neurons that innervate skin and organs (Lumpkin and Caterina, 2007). Among these sensations and perceptions, understanding of the transduction of temperature and heat remains elusive. It has been hypothesized that a stimulus such as heat may require more than one transduction mechanism for thermoperception to occur (Lumpkin and Caterina, 2007). Thermoregulation is a crucial, homeostatic function that promotes an organism’s ability to maintain its core body temperature within a specific range (Hardy, 1961; Hensel, 1973). Changes in the environmental temperature have a more significant and immediate effect on skin temperature, compared to the body’s core temperature (Schlader et al., 2011). This characteristic indicates that changes in the core body temperature cannot necessarily serve as the primary thermal signal. A straightforward example is that the human hand has evolved to perceive highly acute stimuli, with the threshold for detecting a change in temperature being <0.5°C, even if this is not a noxious change (Stevenson et al., 1999; Frenzel et al., 2012). Therefore, the thermoregulatory responses must be rapid, in order to prevent body core temperature challenges (Nakamura, 2011; Schlader et al., 2011), which suggests rapid modifications of the transduction apparatus by signaling molecules.

Transient receptor potential channels are ubiquitous ligand-gated ion channels, known for their role in the detection and perception of various sensations such as pain, pressure, and temperature (Montell and Rubin, 1989; Caterina et al., 1997; Patapoutian et al., 2003), and are, therefore, involved in many important physiological processes (e.g., perception of various stimuli and maintaining ion homeostasis) (Hansen et al., 2001). Eleven TRP channels have been categorized as temperature-sensitive TRPs. Six out of the eleven channels within this category have highly specific temperature thresholds and are known as thermoreceptor TRPs, or “thermoTRPs” (Montell et al., 2002; McKemy, 2005; Tominaga, 2006; Caterina, 2007). TRPV (vanilloid) cation channels 1–4 are thermoTRP channels that are able to detect thermal stimuli ranging from mild warmth to painfully hot (Clapham, 2003; Caterina, 2007; Nakamura and Morrison, 2008; Sisignano et al., 2014). TRPV1 has a temperature threshold of ∼42°C, TRPV2 responds to temperatures ≥52°C, and TRPV3 and TRPV4 are responsive to innocuous warmth (∼26–34°C) (Caterina et al., 1999; Smith et al., 2002; Julius, 2013). ThermoTRP channels exist as membrane proteins that enable ionic mobilization upon stimulation due to a change in temperature (Caterina et al., 1997; Story et al., 2003). If the magnitude of influx is above the threshold, the influx of these ions leads to a temporary depolarization of the cell membrane and initiates a signaling cascade to ultimately allow an organism to perceive that stimulus (Hirst et al., 2006).

The TRPV1 ion channel is the most widely studied receptor of those belonging to the TRPV family. TRPV1 was initially identified in rat dorsal root ganglion (DRG) in 1997, and coined with the name “the capsaicin receptor” (Caterina et al., 1997). These receptors are localized most notably in primary afferent nociceptive neurons in the peripheral nervous system, though they are also expressed in several non-neuronal cell types, along with higher regions of the brain, such as the striatum, hippocampus, midbrain, cerebellum, thalamus, and the hypothalamus (Caterina et al., 1997; Holzer, 2008; Starowicz et al., 2008). The TRPV1 moniker, “the capsaicin receptor,” derived from the receptors’ sensitivity to capsaicin, the primary pungent ingredient in hot chili peppers that elicits a “burning sensation” upon consumption (Jancso et al., 1977). The “burning pain” perception evoked upon encountering capsaicin led to the theory that, due to receptor activation by capsaicin and subsequent response mechanisms determined by the CNS, capsaicin might be eliciting the perception of pain in a manner similar to the endogenous ligands produced and released upon tissue damage (Bevan and Szolcsanyi, 1990; Caterina and Julius, 2001). This drives that hypothesis that there are endogenous ligands that regulate the function of TRPV1.

The discovery of the cannabinoid receptor 1 (CB_1_) lead to the subsequent uncovering of the endocannabinoid (eCB) system. The two most studied signaling molecules within this network are Anandamide (*N*-arachidonoyl ethanolamine; AEA) and 2-arachidonoyl-*sn*-glycerol (2-AG) (Devane et al., 1992; Sugiura et al., 1995). AEA and 2-AG are both derived from membrane phospholipids that contain arachidonic acid and are hypothesized to be produced and released in response to various homeostatic disruptions (Maccarrone et al., 2000). AEA and 2-AG are recognized as lipid signaling molecules that modulate various physiological processes within the nervous system, in part by targeting the cannabinoid receptors, CB_1_ and CB_2_ (Di Marzo and Maccarrone, 2008). Several other members of the *N*-acyl ethanolamine (NAE) family have been identified and appear to be co-regulated by similar biosynthetic and metabolic pathways as AEA, but are conjugated to other fatty acids, such as docosahexaenoic acid, linoleic acid, oleic acid, stearic acid, or palmitic acid (Movahed et al., 2005; Dalle Carbonare et al., 2008). Although AEA was originally identified as an agonist for cannabinoid receptors, it is also capable of activating specialized, non-cannabinoid receptors, as are some of its congeners (Zygmunt et al., 1999; Smart et al., 2000; Ahern, 2003; Movahed et al., 2005). One example of these “non-cannabinoid” receptors are members of the transient receptor potential (TRP) channel superfamily (Raboune et al., 2014).

*N*-arachidonoyl ethanolamine (AEA) is a shared ligand between CB_1_ and TRPV1, synthesized intracellularly in a PKC-mediated, calcium-dependent manner within the nervous system (Okamoto et al., 2007, 2009; Liu et al., 2008). Although not as potent as capsaicin, AEA is considered a full agonist at human TRPV1, as it displays the same maximal effect as capsaicin when administered exogenously in functional assays (Zygmunt et al., 1999; Smart et al., 2000). Additionally, it has been proposed that the activity of AEA at TRPV1 may be dependent on specific ambient temperatures, in a manner similar to capsaicin (Smart et al., 2000; Sprague et al., 2001). An earlier study suggested that 2-AG may have some effect on TRPV1 activity; but as a modulator of membrane potential, rather than as a driver of calcium mobilization (Zygmunt et al., 2013). As an agonist of CB_1_, it is also possible that 2-AG may be involved in the crosstalk between CB_1_ and TRPV1, given the widespread tissue types that co-express these two receptors (Woo et al., 2008). Previously, we showed that capsaicin-stimulated TRPV1-transfected HEK cells drive an increase in production of 2-AG, providing further evidence of a molecular signaling link between the two (Leishman et al., 2017).

Here, we test the hypothesis TRPV1 activity plays an important modulatory role in the biosynthesis and metabolism of AEA, 2-AG and related lipids by analyzing their levels in TRPV1-transfected HEK cells stimulated by either capsaicin or by changes in temperature. We show that there are time and dose dependent effects with capsaicin stimulation and that many of these are mirrored with changes in temperature. Specifically, levels of 2-acyl glycerols, including 2-AG, increased and levels of NAEs, including AEA, decreased with increasing levels of capsaicin and temperature. The opposite effect in lipid production was found when TRPV1-HEK cells were cooled to 27°C in that levels of 2-acyl glycerols decreased and NAEs increased. These data provide a novel insight into the signaling mechanisms of TRPV1 activity and how this signaling system is linked to the eCB signaling system through the modulation of eCB endogenous ligands.

## Materials and Methods

### Analysis of Lipids in TRPV1-HEK Cells After Stimulation by 100 nM, 1 μM, and 10 μM Capsaicin

hTRPV1-HEK cells, a kind gift from Merck (Whitehouse Station, NJ, United States), were grown in high-glucose Dulbecco’s Modified Eagle’s Medium (DMEM) (Thermo Fisher Scientific, Waltham, MA, United States), supplemented with 10% fetal bovine serum (Gibco, Dublin, Ireland), and 1% penicillin/streptomycin antibiotic (Thermo Fisher Scientific), as previously described (Leishman et al., 2017). Stock solutions of capsaicin were prepared in-house in 100% ethanol and stored at −80°C. Solutions of appropriate molarities were made immediately before experimentation in >99% DMSO. For each experiment, TRPV1-HEK cells at approximately 85% confluency were split into 12 individual T-25 cm^2^ flasks. For each condition, the cells were gently washed twice with HEPES buffer [HEPES 0.025 mol, NaCl 0.140 mol, KCl 0.0027 mol, CaCl_2_ 0.0018 mol, MgCl_2_ 0.0005 mol, NaH_2_PO_4_ and D-glucose 0.05 mol (Sigma-Aldrich) in DI H_2_O (prepared in-house)] that had been pre-warmed to 37°C in a water bath. The cells then incubated at 37°C, 5% CO_2_ for 45 min in 4 mL of HEPES buffer. After the 45-min equilibration incubation, the buffer was replaced with 4 mL of the vehicle (0.1% DMSO) or capsaicin-buffer solution. The cells incubated in the capsaicin or vehicle solution for either 1 min or 5 min before lipid extraction was performed. HEPES was used to simulate our calcium-imaging protocols that validated that these cells drive calcium mobilization as previously published (Raboune et al., 2014).

After each flask’s incubation period, the buffer solution was collected in a 15 mL centrifuge tube. Then, 2 mL of 100% HPLC-grade methanol (MeOH; Avantor Performance Materials, Inc., Center Valley, PA, United States) was added to the flask. The cells were harvested from the flasks using cell scrapers, and the cell solution gathered was added to the corresponding collected buffer. An additional 2 mL of MeOH was added to the flask to collect any remaining cells, which were also transferred to the 15 mL centrifuge tube. All tubes were spiked with 50 pmols deuterium-labeled *N*-arachidonoyl glycine (d_8_NaGly; Cayman Chemical, Ann Arbor, MI, United States), prepared in MeOH, which served as an internal standard. After centrifugation, the supernatants were partially purified using C18 solid phase extraction columns (Agilent, Palo Alto, CA, United States) as previously described (Leishman et al., 2018).

### Analysis of Lipids in TRPV1-HEK Cells After Changes in Temperature

60 mm × 15 mm glass petri dishes (Thermo Fisher Scientific) were coated with 50 μg/mL PDL prior to experimentation, to enhance cell adherence. Growth and maintenance of cells was as described above. Cells were grown to 85% confluence before experimentation.

#### Procedure for Temperature Ramp From 27 to 37°C

Cells in the temperature change condition were washed twice with HEPES-Tyrode buffer that had been pre-warmed to 27°C. The cells then incubated in 4 mL HEPES buffer at room temperature inside a cell culture hood for 45 min. In the hood, the cells were covered by a deep stainless-steel tray, accompanied by a beaker of hot water to provide humidity and assist in maintaining a steady temperature of 27°C, which was monitored by a digital wire probe thermometer. After the 45-min incubation period, each dish was transferred to a digital hot plate that had been pre-warmed to 27°C. The lid for the dish was removed and replaced by foil, which was fit snugly over the dish to ensure minimal heat loss and/or evaporation. A wire probe digital thermometer was positioned under the foil and submerged in the buffer solution approximately 2–3 mm from the cell layer to monitor the temperature change. The probe was situated this close in order to avoid mechanical stimulation, but close enough that the temperature being measured was as close as possible to the cell layer. The hot plate temperature was slowly raised using a manual dial while the temperature read-outs from the plate and the probe were monitored, ensuring a steady increase of 15 ± 2–4 s per degree. On average, it took 2.5 min for the buffer solution in each dish to reach 37°C. Upon reaching 37°C, the dish was removed from the hot plate, and the buffer solution was transferred to a conical tube. Subsequently, 2 mL of 100% HPLC-grade MeOH was added to the dish. The cells were then harvested from the dishes using cell scrapers and the cell solution was added to the tube containing the corresponding buffer solution. An additional 2 mL of MeOH was added to the dish to collect any remaining cells, which were also transferred to the conical tube.

#### Procedure for Temperature Ramp From 37 to 45°C

TRPV1-HEK cells were washed twice with HEPES buffer that had been pre-warmed to 37°C. The cells were then incubated at 37°C, 5% CO_2_ for 45 min in 4 mL of HEPES buffer, to allow for equilibration. After the 45-min incubation period, each dish was removed from the incubator and placed on a digital hot plate that was pre-warmed to 37°C. The lid for the dish was removed and replaced with a piece of aluminum foil. A wire probe digital thermometer was positioned under the foil and submerged in the buffer solution to monitor the temperature change. The hot plate temperature was slowly raised, while the temperature read-outs from the plate and the probe were monitored to ensure a steady increase of 15 ± 2–4 s per degree. On average, it took 2–2.25 min for each plate to reach 45°C. Upon reaching 45°C, the dish was removed from the hot plate and the buffer solution was transferred to a conical tube. Cells were harvested from each dish using HPLC-grade MeOH, as described above.

The cells in the control condition for each temperature ramp group were washed twice with HEPES-Tyrode buffer, which was pre-warmed to 37°C, and were then incubated under standard cell culture incubator conditions in 4 mL HEPES buffer for 45 min. The temperature of the solution in each dish was taken after each 45-min incubation period, to ensure the temperature had been maintained at 37°C, ±1°C. The buffer solution was then transferred to a 15 mL conical centrifuge tube. Cells were harvested from each dish using HPLC-grade MeOH, as described above for the temperature-change condition.

To test the hypothesis that the effects of the change in temperature were dependent on TRPV1 activation, cells were treated with 500 nM of the TRPV1 antagonist, iRTX (iodo-resiniferatoxin) prior to the 45-min incubation period and then the identical procedure as outlined above was performed.

All cell solutions from each condition were spiked with 50 pmols d_8_ N-arachidonoyl glycine (NAGly), which served as an internal standard. The spiked samples were centrifuged at 3,000 rpm for 15 min at 24°C. After centrifugation, the supernatants were partially purified using C18 solid phase extraction columns as previously described (Leishman et al., 2018).

### HPLC/MS/MS Quantification and Analysis

Analysis of the samples was carried out using an Applied Biosystems API 3000 triple quadrupole mass spectrometer with electrospray ionization (Applied Biosystems Sciex, Foster City, CA, United States) in MRM mode as previously described (Leishman et al., 2017, 2018). Analyst 1.4.2 software (Applied Biosystems) was used to quantify the HPLC/MS/MS data. The software generated chromatograms by determining the retention times of each analyte with a programmed parent ion and a fragmentation ion mass. Quantification of the analytes were calculated by coupling the calibration curves of the synthetic standards and the recovery adjustments, determined by the d_8_NAGly standard.

Concentrations of each analyte were reported as moles per gram, after measuring the weights of each dried cell pellet. Recoveries were calculated by measuring the amount of d_8_NaGly remaining in each sample after lipid extraction and comparing those values to the recovery standard that represented 100% recovery. IBM SPSS Statistics 23 (IBM, Armonk, NY, United States) was used to determine statistical differences between conditions, implementing one-way ANOVAs. Data comparisons from the capsaicin incubations were made across all three conditions (vehicle, 1-min incubations, and 5 min incubations). A 95% confidence interval was calculated for the means, and statistical significance was defined as *p* ≤ 0.05. For temperature experiments, comparisons were made between the vehicle conditions and the heat-challenged conditions using SPSS one-way ANOVAs. A 95% confidence interval was calculated for the means, and statistical significance was defined as *p* ≤ 0.05. GraphPad Prism 7.0 (GraphPad, San Diego, CA, United States) was used to generate graphs.

## Results

### Lipidomics Analysis of TRPV1-HEK Cells After Capsaicin Stimulation

The following lipids were detected and analyzed in all samples: 2-arachidonoyl glycerol (2-AG), 2-linoleoyl glycerol (2-LG), 2-oleoyl glycerol (2-OG), 2-palmitoyl glycerol (2-PG), AEA, *N*-linoleoyl ethanolamine (LEA), *N*-oleoyl ethanolamine (OEA), *N*-palmitoyl ethanolamine (PEA), *N*-stearoyl ethanolamine (SEA), and *N*-docosahexaenoyl ethanolamine (DEA). Data are analyzed by time; however, they are presented independently by concentration of capsaicin and not as a direct comparison of concentrations. This is because these are data from independent experiments and not on the same cells, which precludes the ability for reliable direct comparisons of absolute lipid concentrations with capsaicin concentrations.

#### Capsaicin-Induced Changes in Lipids Levels After TRPV1-HEK Cells Incubated in 100 nM Capsaicin for 1 min or 5 min

**FIGURE 1:**
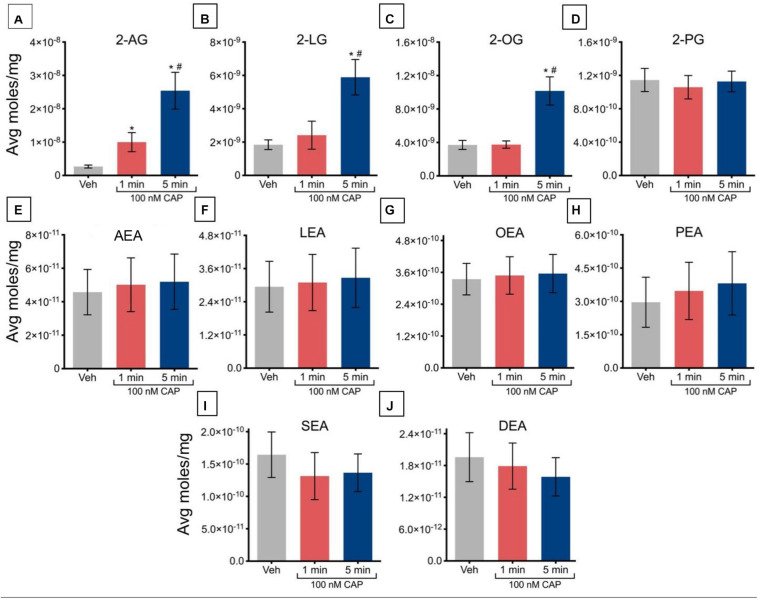
Changes in lipid levels of 2-acyl glycerols **(A–D)** and *N*-acyl ethanolamines **(E–J)** after TRPV1-HEK293 cells incubated in 100 nM capsaicin (CAP) for 1 min or 5 min. Significant changes in lipid levels when comparing capsaicin-induced changes after 1 min or 5 min to vehicle is indicated by * (*p* ≤ 0.05). Significant changes in lipid levels when comparing capsaicin-induced changes after 1 min of incubation vs. 5 min of incubation is indicated by # (*p* ≤ 0.05). Data were averaged over 12 total data points per condition (*n* = 3 individual experiments) and presented as average moles/mg (*y*-axis).

Incubation of TRPV1-HEK293 cells with 100 nM capsaicin for 1 min caused a significant increase in 2-AG concentrations but had no significant effect on the other 2-acyl-glycerols measured. Incubation with 100 nM capsaicin for 5 min drove significant increases in 2-AG, 2-LG, and 2-OG, relative to vehicle (Figures 1A–C). Levels of 2-AG, 2-LG, and 2-OG were significantly higher after 5 min than after 1 min, indicating that regulation of these lipids may be time-dependent at this concentration of capsaicin (Figures 1A–C). There were no significant changes seen in levels of 2-PG at both 1 min and 5 min (Figure 1D). Likewise, no changes were measured in any of the NAEs at either time point (Figures 1E–J).

#### Capsaicin-Induced Changes in Lipids Levels After TRPV1-HEK Cells Incubated in 1 μM Capsaicin for 1 min or 5 min

**FIGURE 2:**
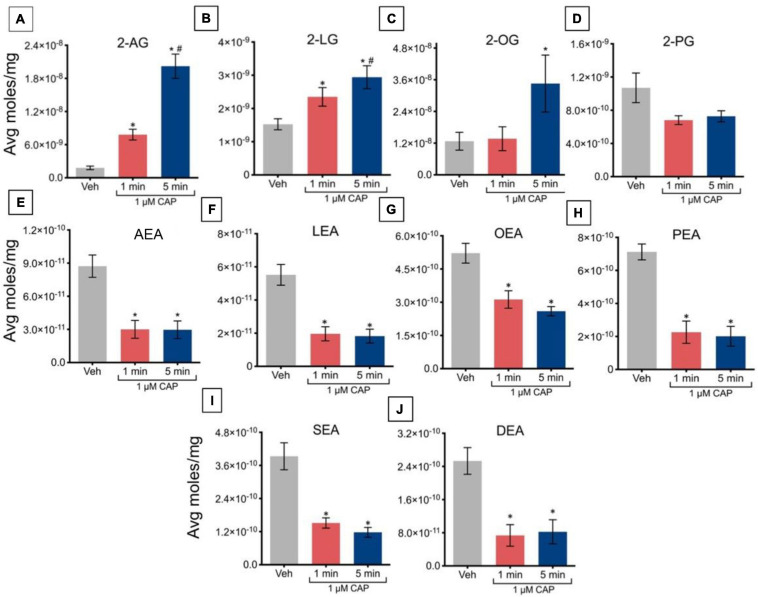
Changes in lipid levels of 2-acyl glycerols **(A–D)** and *N*-acyl ethanolamines **(E–J)** after TRPV1-HEK293 cells incubated in 1 μM capsaicin (CAP) for 1 min or 5 min. Significant changes in lipid levels when comparing capsaicin-induced changes after 1 min or 5 min to vehicle is indicated by * (*p* ≤ 0.05). Significant changes in lipid levels when comparing capsaicin-induced changes after 1 min of incubation vs. 5 min of incubation is indicated by # (*p* ≤ 0.05). Data were averaged over 8 total data points per condition (*n* = 2 individual experiments) and presented as average moles/mg (*y*-axis).

Incubation of TRPV1-HEK293 cells with 1 μM capsaicin for 1 min caused a significant increase in levels of 2-AG and 2-LG, relative to vehicle, with no changes in levels of 2-OG or 2-PG (Figures 2A–D). Capsaicin incubation for 5 min significantly increased levels of 2-AG, 2-LG, and 2-OG, but not 2-PG, relative to vehicle. The increased levels of 2-AG and 2-LG after 5 min of incubation was significantly higher than levels at 1 min, indicating that regulation of these lipids is time-dependent at this concentration of capsaicin. Incubation of TRPV1-HEK293 cells with 1 μM capsaicin for 1 min and 5 min significantly decreased levels of all NAEs, including the eCB anandamide. In contrast to effects on 2-AG and 2-LG, which were time-dependent, the effects on NAEs were independent of time, showing similar decreases at both time points (Figures 2E–J).

#### Capsaicin-Induced Changes in Lipids Levels After TRPV1-HEK Cells Incubated in 10 μM Capsaicin for 1 min or 5 min

**FIGURE 3:**
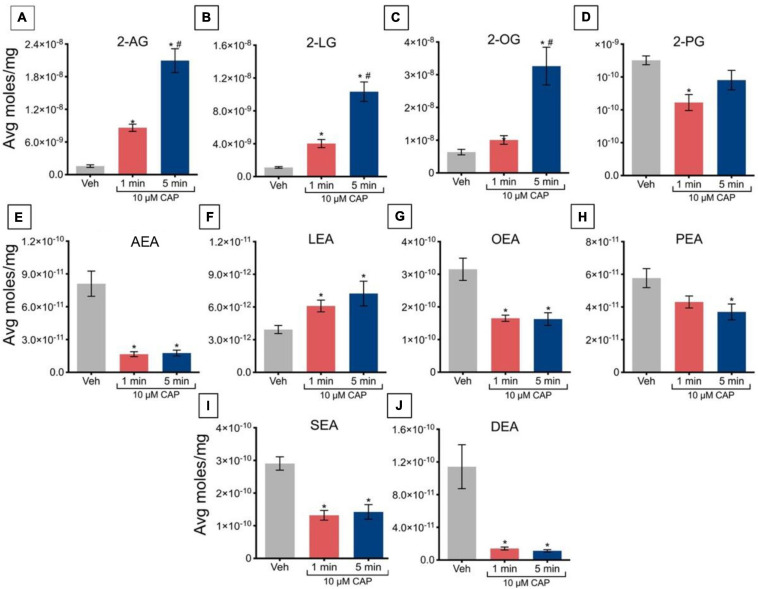
Changes in lipid levels of 2-acyl glycerols **(A–D)** *N*-acyl ethanolamines **(E–J)** after TRPV1-HEK293 cells incubated in 10 μM capsaicin (CAP) for either 1 min or 5 min. Significant changes in lipid levels when comparing capsaicin-induced changes after 1 min or 5 min to vehicle is indicated by * (*p* ≤ 0.05). Significant changes in lipid levels when comparing capsaicin-induced changes after 1 min of incubation vs. 5 min of incubation is indicated by # (*p* ≤ 0.05). Data depicted in each graph were averaged over 16 total data points per condition (*n* = 4 individual experiments) and presented as average moles/mg (*y*-axis).

Incubation of TRPV1-HEK293 cells with 10 μM capsaicin for 1 min significantly increased levels of 2-AG, 2-LG, and 2-OG, relative to vehicle (Figures 3A–C); however, this incubation significantly decreased levels of 2-PG (Figure 3D). At 5 min, levels of 2-AG, 2-LG, and 2-OG remained elevated relative to vehicle, but no significant changes were observed in the levels of 2-PG. Effects on 2-acyl glycerols were time-dependent, with significantly higher levels of 2-AG, 2-LG, and 2-OG after 5 min of incubation versus 1 min. Incubation of TRPV1-HEK293 cells with 10 μM capsaicin for 1 min and 5 min significantly reduced levels of AEA, OEA, SEA, and DEA, relative to vehicle (Figures 3E,G,I,J), whereas LEA levels increased relative to vehicle (Figure 3F), while levels of PEA remained unchanged (Figure 3H). No significant effects of time were detected for NAEs in these experiments, in that levels of NAEs did not further change at 5 min versus 1 min (Figures 3E–J).

### Lipidomics Analysis of TRPV1-HEK Cells After Changes in Temperature

#### Temperature-Induced Changes in Lipid Levels After Change From 27 to 37°C in TRPV1-HEK Cells

**FIGURE 4:**
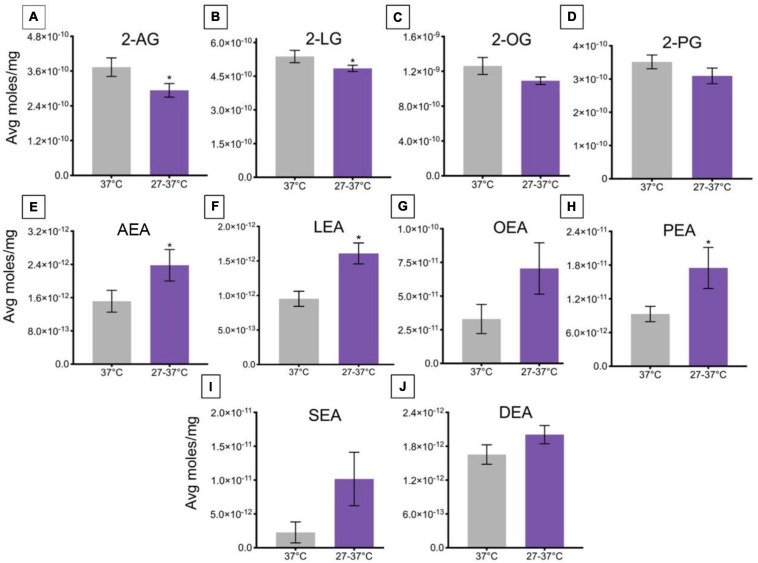
Changes in lipid levels of 2-acyl glycerols **(A–D)** and *N*-acyl ethanolamines **(E–J)** upon an increase in temperature from 27 to 37°C. Significant temperature-induced changes after TRPV1-HEK293 cells were exposed to a change in ambient temperature from 27 to 37°C is indicated by * (*p* ≤ 0.05). Data depicted in each graph were averaged over 15 total data points per condition (*n* = 3 individual experiments) and are presented as average moles/mg (*y*-axis).

A change in ambient temperature to 27°C for 45 min and then a ramp to 37°C over ∼2 min caused a significant decrease the levels of 2-AG and 2-LG, relative to controls maintained at a temperature of 37°C (Figures 4A,B). Levels of 2-OG and 2-PG were unaffected by this change in temperature (Figures 4C,D). This change in temperature drove a significant increase in levels of AEA, LEA, and PEA, relative to a maintained temperature of 37°C (Figures 4E,F,H), whereas no significant changes occurred in levels of OEA, SEA, and DEA (Figures 4G,I,J).

#### Temperature-Induced Changes in Lipid Levels After Change From 37 to 45°C in TRPV1-HEK Cells

**FIGURE 5:**
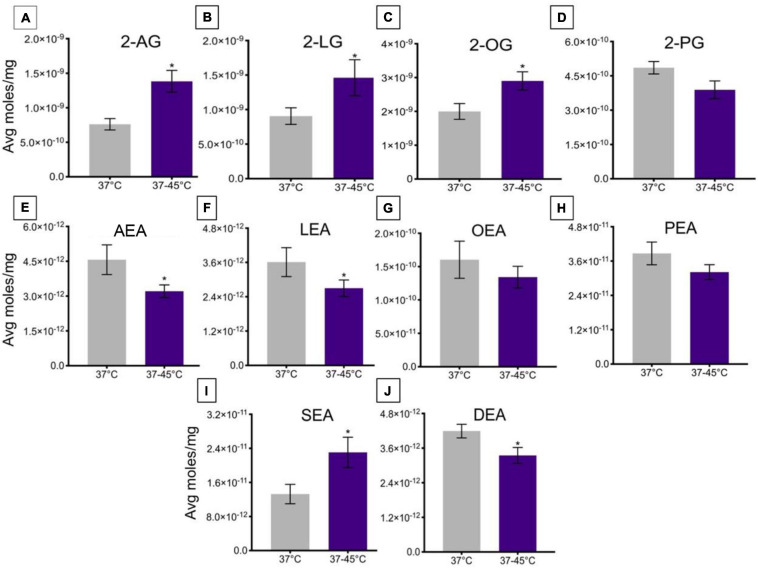
Changes in lipid levels of 2-acyl glycerols **(A–D)** and *N*-acyl ethanolamines **(E–J)** upon an increase in temperature from 37 to 45°C. Significant temperature-induced changes after TRPV1-HEK293 cells were exposed to a change in ambient temperature from 37 to 45°C, is indicated by * (*p* ≤ 0.05). Data depicted in each graph were averaged over 15 total data points per condition (*n* = 3 individual experiments) and are presented as average moles/mg (*y*-axis).

A change in ambient temperature from 37 to 45°C over ∼2.5 min caused a significant increase in levels of 2-AG, 2-LG, and 2-OG (Figures 5A–C), whereas there was no significant change in levels of 2-PG (Figure 5D). This change in temperature significantly decreased levels of AEA, LEA, and DEA (Figures 5E,F,J), whereas the change in temperature caused a significant increase in lipid levels of SEA (Figure 5I). No significant changes were measured in levels of OEA or PEA (Figures 5G,H).

#### Temperature-Induced Changes in Lipid Levels After Change From 37°C–45°C in iRTX-Treated TRPV1-HEK Cells

To determine the extent to which TRPV1 activation during heat ramp stimulation drives changes in these lipids the same testing protocol was used on HEK TRPV1 cells with a pre-treatment of 500 nM iRTX, a potent TRPV1 antagonist. Table 1 shows that there is only one significant change in any of the lipids measured. 2-LG was still significantly increased in the presence of iRTX; however, the magnitude of effect is much reduced. These data support the involvement of TRPV1 in the modulation of these lipids.

**TABLE 1:** Levels of lipids in HEK-TRPV1 cells after heat ramp from 37–45°C in the presence of the TRPV1 antagonist, iRTX.

	**500 nM iRTX**	**Mean**	**SEM**
2-AG	37°C	3.07E-10	2.25E-11
	37–45°C	3.39E-10	3.40E-11
2-LG	37°C	3.43E-10	2.34E-11
	37–45°C	**4.44E-10**	3.00E-11
2-OG	37°C	5.88E-09	2.45E-10
	37–45°C	6.90E-09	5.22E-10
2-PG	37°C	4.56E-10	3.63E-11
	37–45°C	4.69E-10	3.71E-11
AEA	37°C	7.56E-11	6.31E-12
	37–45°C	6.18E-11	5.26E-12
LEA	37°C	6.53E-10	3.87E-11
	37–45°C	7.40E-10	3.32E-11
OEA	37°C	5.16E-11	4.01E-12
	37–45°C	6.01E-11	3.02E-12
PEA	37°C	2.73E-10	1.87E-11
	37–45°C	3.21E-10	2.04E-11
SEA	37°C	4.04E-10	2.77E-11
	37–45°C	3.56E-10	2.99E-11
DEA	37°C	1.83E-10	1.22E-11
	37–45°C	2.27E-10	2.05E-11

*All lipids levels are expressed as mols/gram dried cell pellet weight. Bold indicates *p* < 0.05. See text for abbreviations.*

## Discussion

The detection of ambient temperature is vital for organisms that seek temperatures that are optimal for life, and to avoid any potentially damaging environmental conditions. The activity of thermoTRPs facilitates the ability of organisms to adjust other physiological functions and behaviors, upon detecting an environmental cue such as temperature (Castillo et al., 2018). This type of regulation can ensure that prolonged periods of core body temperature elevation or depression are minimized, preserving a hospitable environment for necessary enzymatic reactions to occur (Romanovsky, 2007). As a thermoTRP, a notable characteristic of TRPV1 channels is that they are responsive to exogenous and endogenous chemical stimuli (capsaicin) and physical stimuli, such as heat (Caterina et al., 1997; Clapham, 2003). This characteristic ability to respond to such a range of stimuli categorizes TRPV1 as a polymodal receptor. As a polymodal molecular integrator, TRPV1 has evolved an ability to have several mechanisms/pathways by which it can transduce information gathered from a diverse set of ligands (Julius and Basbaum, 2001; Raboune et al., 2014). Our current data suggests that TRPV1 activation drives changes in eCB and related lipid mediators by both chemical and thermal stimuli. These data presented in this study provide a novel insight into the dynamic molecular signaling systems involved in the thermoregulatory system at the level of TRPV1 activation.

The surprising finding that increases in levels of 2-AG corresponded with decreases in levels of AEA with both capsaicin and heat in a time range of only 1–5 min suggests a potential molecular link in their biosynthesis and/or metabolism. It is a typical assumption when measuring an increase in a signaling molecule to infer that the metabolism has been inhibited. Likewise, there is often the inference of an upregulation in metabolism when the levels of a molecule decrease. The other side of the equation rests with the regulation of the biosynthesis wherein increases in a molecule can denote an upregulation of this biosynthesis process and a downregulation with decreases. Both processes may also be at play in the rapid modulation to a cellular event, especially when two divergent pathways for biosynthesis share a common precursor.

While AEA and 2-AG both contain arachidonic acid (AA), their biosynthetic pathways are not through conjugation of free AA; instead, they are both produced through enzymatic modulation of AA-containing membrane phospholipids (Liu et al., 2008; Okamoto et al., 2009; Leishman et al., 2016; Mock et al., 2020). Three separate synthesis pathways have been hypothesized to generate AEA (Ueda et al., 2013). Each of these pathways require availability of *N*-arachidonoyl-phosphatidylethanolamine (NAPE). NAPE is the product of the membrane phospholipid, phosphatidylethanolamine (PE) through an acyl transfer from phosphatidylcholine (PC) via an *N*-acyl transferase (NAT) enzyme in a calcium-dependent manner (Di Marzo et al., 1994). More recently, the Cravatt group isolated the serine hydrolase PLA2G4E as a specific NAT in CNS tissue that produces the NAPE species that are precursors to NAEs including AEA (Ogura et al., 2016). NAPE-PLD cleaves the NAPE glycerophosphate group via hydrolysis, yielding NAEs (Di Marzo et al., 1994; Leishman et al., 2016). The second pathway first requires conversion of NAPE into lyso-NAPE, which occurs when the two NAPE-specific phospholipases (A1/A2) cleave the molecule into lyso-NAPE and glycerophosphoanandamide. From here, a specific PLD can hydrolyze the intermediate molecule to yield NAEs (Simon and Cravatt, 2006). The third pathway requires the use of a NAPE-specific PLC to cleave NAPE and acquire phospho-NAE. Dephosphorylation of this intermediate using a lipid phosphatate yields NAEs (Liu et al., 2008).

Biosynthesis of 2-AG is like AEA in that both are derived from membrane phospholipids. Diacylglycerols (DAGs) are analogous to NAPEs in that they are likewise generated by multiple pathways (Stella et al., 1997; Sugiura et al., 2006). One of the pathways to generate DAGs is through the hydrolysis of phosphatidic acid (PA), which can be generated from PC enzymatically via phospholipase D (PLD) or phospholipase C (PLC), and a third is through the direct hydrolysis of triacylglycerols (Stella et al., 1997; Sugiura et al., 2006). DAGs are then hydrolyzed via diacylglycerol lipase (DAGL) into monoacylglycerols (MAGs) such as the 2-acyl glycerols including the eCB, 2-AG (Sugiura et al., 2006).

**FIGURE 6:**
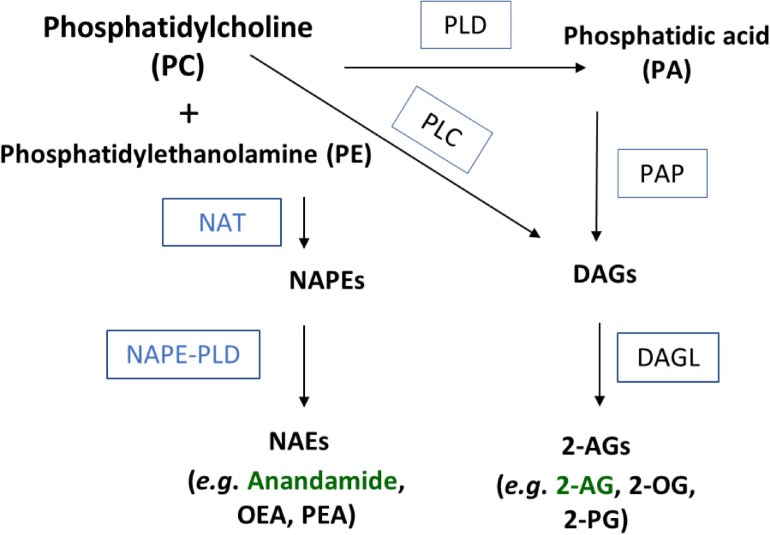
Schematic of NAE and 2-acyl glycerol biosynthesis through a phosphatidylcholine (PC) precursor. Substrates and products in bold black. Enzymes abbreviated in boxes. NAT, N-acyl transferase; PLD, phospholipase D; PLC, phospholipase C; PA, phosphatidic acid; NAPE, N-acyl phosphatidylethanolamine; PAP, phosphatidic acid phosphohydrolase; DAG, diacylglycerol; DAGL, diacylglycerol lipase.

It is important to note that PC is a precursor to both AEA and 2-AG. We hypothesize that the regulation of PC metabolism may be a factor in shunting toward one pathway over the other. The evidence that lower temperatures drive the opposite effect with increases and NEAs and decreases in 2-acyl glycerols further supports this hypothesis. Figure 6 outlines the specific pathways that involve PC in both NAE and 2-acyl glycerol biosynthesis. We hypothesize that TRPV1 activity is driving a shift in the amount of PC being shunted in the PLD pathway toward DAGs, which is creating a precursor deficit for NAPE and then NAE production. As with all hypotheses, this one comes with caveats. While AEA and 2-AG as well as OEA and 2-OG have a very predictable inverse relationship with TRPV1 activity at 1 and 10 μM capsaicin and heat, the linoleoyl derivatives (2-LG and LEA) only show this tight relationship with 1 μM capsaicin and heat. Whereas PEA and 2-PG do not show this same relationship, wherein 2-PG and PEA are less effected by capsaicin and heat overall, but both decrease with 10 μM capsaicin. The standards for 2-stearoyl glycerol and 2-docosahexaenoyl glycerol were not available for analysis during these experiments, so we were unable to ascertain the relationships between the species. However, it is important to note that SEA increased with heat instead of decreased like the rest of the NAEs; therefore, it is likely that there are multiple ways that these signaling molecules are regulated with temperature. What the purpose of these lipid signaling molecules is another important aspect of study for future experiments.

## Conclusion

Here, we demonstrate that activity of TRPV1 via capsaicin and temperature drives increases in levels of 2-acyl glycerols and decreases in NAEs in a time and dose dependent manner. These novel findings provide a unique insight in the signaling systems involving TPRV1 activity and in lipid signaling molecule regulation by temperature. The data further illustrate that the modulation of intracellular calcium is only one of the many signaling cascades that are relevant to TRPV1 signaling.

## Data Availability Statement

The raw data supporting the conclusions of this article will be made available by the authors, without undue reservation.

## Author Contributions

MM, KS, and AA performed the TRPV1 HEK cell experiments (MM and KS with capsaicin, MM and AA with temperature). EL performed the mass spectrometric analysis. MM and KS performed the statistical analyses. MM, EL, and HB shared contribution to the manuscript. MM and HB conceived of the idea and developed methods for experimental implementation. All authors contributed to the article and approved the submitted version.

## Conflict of Interest

HB is on the scientific advisory board for PhytECS and Medicane. Neither company had any financial relationship to this or any research in the Bradshaw law. The remaining authors declare that the research was conducted in the absence of any commercial or financial relationships that could be construed as a potential conflict of interest.
